# Risk Prediction for Breast, Endometrial, and Ovarian Cancer in White Women Aged 50 y or Older: Derivation and Validation from Population-Based Cohort Studies

**DOI:** 10.1371/journal.pmed.1001492

**Published:** 2013-07-30

**Authors:** Ruth M. Pfeiffer, Yikyung Park, Aimée R. Kreimer, James V. Lacey, David Pee, Robert T. Greenlee, Saundra S. Buys, Albert Hollenbeck, Bernard Rosner, Mitchell H. Gail, Patricia Hartge

**Affiliations:** 1 Division of Cancer Epidemiology and Genetics, National Cancer Institute, National Institutes of Health, Bethesda, Maryland, United States of America; 2 City of Hope, Duarte, California, United States of America; 3 Information Management Systems, Rockville, Maryland, United States of America; 4 Marshfield Clinic Research Foundation, Marshfield, Wisconsin, United States of America; 5 Department of Internal Medicine, University of Utah Health Sciences Center, Salt Lake City, Utah, United States of America; 6 AARP, Washington, District of Columbia, United States of America; 7 Channing Laboratory, Brigham and Women's Hospital and Harvard School of Public Health, Harvard University, Boston, Massachusetts, United States of America; McGill University, Canada

## Abstract

**Background:**

Breast, endometrial, and ovarian cancers share some hormonal and epidemiologic risk factors. While several models predict absolute risk of breast cancer, there are few models for ovarian cancer in the general population, and none for endometrial cancer.

**Methods and Findings:**

Using data on white, non-Hispanic women aged 50+ y from two large population-based cohorts (the Prostate, Lung, Colorectal, and Ovarian Cancer Screening Trial [PLCO] and the National Institutes of Health–AARP Diet and Health Study [NIH-AARP]), we estimated relative and attributable risks and combined them with age-specific US-population incidence and competing mortality rates. All models included parity. The breast cancer model additionally included estrogen and progestin menopausal hormone therapy (MHT) use, other MHT use, age at first live birth, menopausal status, age at menopause, family history of breast or ovarian cancer, benign breast disease/biopsies, alcohol consumption, and body mass index (BMI); the endometrial model included menopausal status, age at menopause, BMI, smoking, oral contraceptive use, MHT use, and an interaction term between BMI and MHT use; the ovarian model included oral contraceptive use, MHT use, and family history or breast or ovarian cancer. In independent validation data (Nurses' Health Study cohort) the breast and ovarian cancer models were well calibrated; expected to observed cancer ratios were 1.00 (95% confidence interval [CI]: 0.96–1.04) for breast cancer and 1.08 (95% CI: 0.97–1.19) for ovarian cancer. The number of endometrial cancers was significantly overestimated, expected/observed = 1.20 (95% CI: 1.11–1.29). The areas under the receiver operating characteristic curves (AUCs; discriminatory power) were 0.58 (95% CI: 0.57–0.59), 0.59 (95% CI: 0.56–0.63), and 0.68 (95% CI: 0.66–0.70) for the breast, ovarian, and endometrial models, respectively.

**Conclusions:**

These models predict absolute risks for breast, endometrial, and ovarian cancers from easily obtainable risk factors and may assist in clinical decision-making. Limitations are the modest discriminatory ability of the breast and ovarian models and that these models may not generalize to women of other races.

*Please see later in the article for the Editors' Summary*

## Abstract 2

Ruth Pfeiffer and colleagues describe models to calculate absolute risks for breast, endometrial, and ovarian cancers for white, non-Hispanic women over 50 years old using easily obtainable risk factors.

*Please see later in the article for the Editors' Summary*

## Editors' Summary

**Background:**

In 2008, just three types of cancer accounted for 10% of global cancer-related deaths. That year, about 460,000 women died from breast cancer (the most frequently diagnosed cancer among women and the fifth most common cause of cancer-related death). Another 140,000 women died from ovarian cancer, and 74,000 died from endometrial (womb) cancer (the 14th and 20th most common causes of cancer-related death, respectively). Although these three cancers originate in different tissues, they nevertheless share many risk factors. For example, current age, age at menarche (first period), and parity (the number of children a woman has had) are all strongly associated with breast, ovarian, and endometrial cancer risk. Because these cancers share many hormonal and epidemiological risk factors, a woman with a high breast cancer risk is also likely to have an above-average risk of developing ovarian or endometrial cancer.

**Why Was This Study Done?:**

Several statistical models (for example, the Breast Cancer Risk Assessment Tool) have been developed that estimate a woman's absolute risk (probability) of developing breast cancer over the next few years or over her lifetime. Absolute risk prediction models are useful in the design of cancer prevention trials and can also help women make informed decisions about cancer prevention and treatment options. For example, a woman at high risk of breast cancer might decide to take tamoxifen for breast cancer prevention, but ideally she needs to know her absolute endometrial cancer risk before doing so because tamoxifen increases the risk of this cancer. Similarly, knowledge of her ovarian cancer risk might influence a woman's decision regarding prophylactic removal of her ovaries to reduce her breast cancer risk. There are few absolute risk prediction models for ovarian cancer, and none for endometrial cancer, so here the researchers develop models to predict the risk of these cancers and of breast cancer.

**What Did the Researchers Do and Find?:**

Absolute risk prediction models are constructed by combining estimates for risk factors from cohorts with population-based incidence rates from cancer registries. Models are validated in an independent cohort by testing their ability to identify people with the disease in an independent cohort and their ability to predict the observed numbers of incident cases. The researchers used data on white, non-Hispanic women aged 50 years or older that were collected during two large prospective US cohort studies of cancer screening and of diet and health, and US cancer incidence and mortality rates provided by the Surveillance, Epidemiology, and End Results Program to build their models. The models all included parity as a risk factor, as well as other factors. The model for endometrial cancer, for example, also included menopausal status, age at menopause, body mass index (an indicator of the amount of body fat), oral contraceptive use, menopausal hormone therapy use, and an interaction term between menopausal hormone therapy use and body mass index. Individual women's risk for endometrial cancer calculated using this model ranged from 1.22% to 17.8% over the next 20 years depending on their exposure to various risk factors. Validation of the models using data from the US Nurses' Health Study indicated that the endometrial cancer model overestimated the risk of endometrial cancer but that the breast and ovarian cancer models were well calibrated—the predicted and observed risks for these cancers in the validation cohort agreed closely. Finally, the discriminatory power of the models (a measure of how well a model separates people who have a disease from people who do not have the disease) was modest for the breast and ovarian cancer models but somewhat better for the endometrial cancer model.

**What Do These Findings Mean?:**

These findings show that breast, ovarian, and endometrial cancer can all be predicted using information on known risk factors for these cancers that is easily obtainable. Because these models were constructed and validated using data from white, non-Hispanic women aged 50 years or older, they may not accurately predict absolute risk for these cancers for women of other races or ethnicities. Moreover, the modest discriminatory power of the breast and ovarian cancer models means they cannot be used to decide which women should be routinely screened for these cancers. Importantly, however, these well-calibrated models should provide realistic information about an individual's risk of developing breast, ovarian, or endometrial cancer that can be used in clinical decision-making and that may assist in the identification of potential participants for research studies.

**Additional Information:**

Please access these websites via the online version of this summary at http://dx.doi.org/10.1371/journal.pmed.1001492.

- This study is further discussed in a *PLOS Medicine* Perspective by Lars Holmberg and Andrew Vickers
- The US National Cancer Institute provides comprehensive information about cancer (in English and Spanish), including detailed information about breast cancer, ovarian cancer, and endometrial cancer;
- Information on the Breast Cancer Risk Assessment Tool, the Surveillance, Epidemiology, and End Results Program, and on the prospective cohort study of screening and the diet and health study that provided the data used to build the models is also available on the NCI site
- Cancer Research UK, a not-for-profit organization, provides information about cancer, including detailed information on breast cancer, ovarian cancer, and endometrial cancer
- The UK National Health Service Choices website has information and personal stories about breast cancer, ovarian cancer, and endometrial cancer; the not-for-profit organization Healthtalkonline also provides personal stories about dealing with breast cancer and ovarian cancer

## Introduction

Several statistical models predict a woman's probability or absolute risk of developing invasive breast cancer based on her age and reproductive, medical, and lifestyle factors [1]–[5]. Many risk factors associated with breast cancer are also associated with the risk of other gynecologic cancers. For example, four of the seven risk factors used in the publicly available Breast Cancer Risk Assessment Tool (BCRAT; http://www.cancer.gov/bcrisktool) [6] are also strongly associated with ovarian and endometrial cancer risks, including current age, age at menarche, parity, and first-degree family history of breast cancer. Therefore, a woman with a high breast cancer risk due to combinations of these risk factors likely also has above-average endometrial or ovarian cancer risk. However, while some models have been proposed for risk prediction for ovarian cancer [7], no model that predicts the absolute risk of endometrial cancer is available, even though endometrial cancer is the fourth most common cancer in women, and 1 in 38 women will be diagnosed with endometrial cancer during her lifetime [8]. With rates of obesity increasing and rates of hysterectomy declining in many regions of the US, endometrial cancer incidence may rise further, and thus it is important to identify women at highest risk for this disease.

Absolute risk prediction models provide useful information for health care providers and patients and aid in the design and recruitment phase of studies of preventive interventions. Several large chemoprevention studies of tamoxifen (Nolvadex; AstraZeneca) and raloxifene (Evista; Lilly) used BCRAT projected breast cancer risk to determine eligibility and to estimate needed sample sizes [9],[10]. These models can also aid clinical management of women at elevated risk of one or more of these outcomes. For example, if a woman presents with endometrial bleeding, but is found not to have endometrial cancer in a subsequent workup, an estimate of her risk of developing endometrial cancer over the next 5 or 10 y may aid her and her physician in deciding further steps, including having a hysterectomy or taking progestin. Knowing her risk of endometrial cancer in addition to her risk of breast cancer could also inform a woman's decision about whether or not to consider use of tamoxifen for breast cancer prevention [11], as tamoxifen reduces breast cancer risk, but also increases the risk of endometrial cancer [10]. Additionally, knowing her ovarian cancer risk might influence her decision regarding prophylactic bilateral oophorectomy to reduce her risk of breast cancer.

We developed absolute risk prediction models for breast, endometrial, and ovarian cancer by combining data from two large prospective cohorts at the National Cancer Institute (NCI)—the Prostate, Lung, Colorectal, and Ovarian Cancer Screening Trial (PLCO) and the National Institutes of Health–AARP Diet and Health Study (NIH-AARP)—and from incidence and competing mortality rates in the NCI's Surveillance, Epidemiology, and End Results Program (SEER). The SEER cancer registries cover approximately 28% of the US population (http://seer.cancer.gov/about/overview.html).

All models were validated using independent data from the Nurses' Health Study (NHS).

## Methods

### Data for Relative Risk Models

We used data on white, non-Hispanic women from two NCI cohorts: the PLCO cohort and the NIH-AARP cohort.

#### PLCO

PLCO has been described in detail previously [12]. In brief, PLCO enrolled 78,232 women, aged 55 to 74 y at baseline, between November 1993 and June 2001 at ten screening centers. Women were eligible if they had no history of lung, colorectal, or ovarian cancer and were neither undergoing cancer treatment nor participating in other screening or prevention trials. Women who had undergone bilateral oophorectomy or were taking tamoxifen were initially ineligible but were later included. Women randomized to the screening arm of the trial received a single-view chest X-ray annually for 4 y to screen for lung cancer; a CA (cancer antigen) 125 blood test annually for 6 y and a transvaginal ultrasound yearly for 4 y, both to screen for ovarian cancer; and flexible sigmoidoscopy at the beginning of the trial and either 3 y or 5 y later to screen for colorectal cancer. Institutional review boards at the NCI and screening centers approved the study.

At entry, participants completed a self-administered lifestyle questionnaire. Cancers were ascertained via annual study updates and death certificates, and verified via review of medical records.

#### NIH-AARP

NIH-AARP has been described previously [13]. It included 567,169 men and women who, in 1995–1996, were 50–71 y old and resided in one of eight states. Participants returned a self-administered baseline questionnaire and a second more detailed questionnaire, sent 6 mo after the baseline questionnaire. The NCI Special Studies Institutional Review Board approved the study.

Cancer cases were identified through linkage with state cancer registries with a 90% completeness rate [14]. All cases had a histologic diagnosis. Vital status was ascertained through annual linkage to the Social Security Administration Death Master File and the National Death Index Plus.

#### Analytic populations

In each study, we restricted the analysis to non-Hispanic, white women who completed the baseline questionnaire, had follow-up information, and had no personal history of the cancer of interest at baseline. For the ovarian and endometrial cancer models, we further excluded women with bilateral oophorectomy or hysterectomy, respectively (see Table 1 for further details on exclusions). After these exclusions, the NIH-AARP and PLCO study populations included 191,604 and 64,440 women, respectively, for the breast cancer analysis, 114,931 and 42,821 women, respectively, for the endometrial cancer analysis, and 151,165 and 58,282 women, respectively, for the ovarian cancer analysis.

**Table 1:** Study populations and exclusions for model development: NIH-AARP and PLCO cohorts at baseline.

Cohort Subsets	Breast Cancer Model		Endometrial Cancer Model		Ovarian Cancer Model	
	NIH-AARP	PLCO	NIH-AARP	PLCO	NIH-AARP	PLCO
Total cohort	226,733	78,217	226,733	78,217	226,733	78,217
Proxy response/no baseline questionnaire	1,265	2,095	1,265	2,095	1,265	2,095
Non-white	23,920	8,720	23,920	8,720	23,920	8,720
Organ removed or unknown at baseline	—	—	86,587	24,189	49,956	8,608
No follow-up time after baseline	4	565	3	373	4	505
Prevalent cancer of interest	9,940	2,397	27	19	423	—
Analytic cohort for model building	191,604	64,440	114,931	42,821	151,165	58,289
Cases used for model building	5,870	2,288	1,185	471	597	284
Missing covariates	13,141	2,191	9,946	1,127	7,756	1,725
Final analytic cohort	178,463	62,249	104,985	41,694	143,409	56,564
Final number of cases used in the models	5,480	2,215	1,097	462	570	274

### Data for Model Validation

Independent data from the NHS cohort from July 1990 to June 2004 were used to validate our models. The NHS cohort included 121,701 women aged 30–55 y in 1976 [15]. All participants gave informed consent at enrollment. With the same exclusions as for the relative risk (RR) models, the validations were based on 57,906 women for the breast cancer model and 37,241 for the endometrial cancer model. For the ovarian cancer absolute risk model we additionally excluded women who reported removal of a single ovary or ovarian surgery with unknown status of the ovaries during follow-up, resulting in 56,638 women for validation. See Table 2 for details on exclusions. Breast and ovarian cancer incidence rates were similar to those in SEER, but endometrial cancer incidence was substantially lower, with an overall incidence at age 50–74 y of 42.16 per 100,000 person-years, compared to 78.1 per 100,000 person-years in SEER for the same age range (see Tables 3 and 4 for further details).

**Table 2:** Study population and exclusions for model validation: NHS cohort at baseline.

Cohort Subsets	Breast Cancer Model	Endometrial Cancer Model	Ovarian Cancer Model
Total cohort	121,701	121,701	121,701
Age at entry <50 y	24,366	24,366	24,366
Non-white	3,268	3,268	3,268
Prevalent cancer of interest	2,702	317	277
No follow-up time after baseline	3,710	4,180	4,030
Missing birth year	123	123	123
Organ removed/unknown at baseline	—	32,712	16,512
Missing covariates	29,625	8,750	3,909
Age at exit ≥90 y	1	1	1
Organ removed/unknown during follow-up		10,743	12,577
Analytic cohort	57,906	37,241	56,638
Cases	2,934	532	377

**Table 3:** Age-specific incidence per 100,000 person-years in non-Hispanic white women from the NHS cohort, excluding only women with prevalent cancer of interest and no positive follow-up time.

Age Group	Breast Cancer	Endometrial Cancer^a^	Ovarian Cancer
50–54 y	293.2	40.7	25.1
55–59 y	312.5	42.8	34.1
60–64 y	352.0	40.2	36.3
65–69 y	347.1	45.0	40.8
All	325.5	49.7	34.0

a Cancer of the corpus uteri or uterus, not otherwise specified.

**Table 4:** 5-y age-specific SEER incidence rates, 1992–2006, for breast, endometrial, and ovarian cancers for white, non-Hispanic females in 13 SEER registries (Alaska excluded) that cover 14% of the US population.

Age Group	Total Population	Breast Cancer	Endometrial Cancer^a^	Endometrial Cancer^a^ Corrected for Hysterectomy	Ovarian Cancer	Ovarian Cancer Corrected for Bilateral Oophorectomy
50–54 y	11,228,901	272.42	50.50	76.24	25.93	32.20
55–59 y	9,134,183	338.62	77.50	127.55	33.50	41.61
60–64 y	7,379,976	410.08	97.44	174.72	43.47	59.28
65–69 y	6,692,181	465.29	106.50	193.67	49.06	70.61
70–74 y	6,386,868	499.40	109.02	199.96	56.15	73.12
75–79 y	5,797,382	518.48	106.05	196.35	60.25	81.59
80–84 y	4,460,480	491.45	96.76	172.35	62.68	85.04
85+ y	4,403,964	408.84	69.26	118.09	54.61	65.10

Source: SEER (http://seer.cancer.gov/).

a Cancer of the corpus uteri or uterus, not otherwise specified.

### Statistical Methods

#### Relative risk models

We estimated separate RR models for breast, endometrial, and ovarian cancer using Cox proportional hazards regression (SAS, version 9.1; SAS Institute), with age as the timescale. For each of the cancers, the primary outcome was incident, invasive epithelial cancer. For each outcome we first estimated RRs and 95% confidence intervals (CIs) separately for each cohort. However, we used RRs from a Cox model for the combined cohorts that included a study indicator as covariate (NIH-AARP versus PLCO). Analyses of the combined data that stratified the baseline hazard function in the Cox model on study yielded results in agreement to the third decimal point with this regression method. Proportionality of the hazard functions was assessed by visual inspection of hazard plots and Schoenfeld residuals.

For each RR model, women were considered at risk from the age at study entry (randomization for PLCO and completion of baseline questionnaire for NIH-AARP) until the age at the earliest of the following: (1) diagnosis of cancer of interest, (2) death, or (3) administrative censoring (for PLCO, most recent annual study update through December 31, 2005; for NIH-AARP, December 31, 2003). In PLCO, women were also censored at date of unconfirmed self-reported cancers and non-epithelial cancers (the *International Classification of Diseases for Oncology, Third Edition* [16] morphology codes 8000 to 8573). For the breast cancer RR analysis, women were additionally censored at diagnosis of breast carcinoma in situ, and for the ovarian cancer RR analysis, at diagnosis of an ovarian tumor of low malignant potential in PLCO.

We considered the following risk factors (coding in parentheses): body mass index (BMI; <25, 25 to <30, 30 to <35, 35 to <40, and 40+ kg/m^2^), age at menarche (see comment on coding below), number of live-born children (parity; 0, 1, 2, 3–4, 5–9, 10+), age at first birth (no children or at age <16, 16–19, 20–24, 25–29, 30–34, 35–39, 40+ y), duration of oral contraceptive (OC) use (never or <1, 1–4, 5–9, 10+ y), menopausal status and age at natural menopause (still menstruating or age <40, 40–44, 45–49, 50–54, ≥55 y), status and duration of menopausal hormone therapy (MHT) use (never, current use, former use; 0, <5, 5–9, 10+ y), status and duration of estrogen and progestin MHT use (never, current use, former use; <1, 2, 3, …, 9, 10+ y), duration of unopposed estrogen MHT use (current use, former use, never; <1, 2, 3, …, 9, 10+ y), history of benign breast disease (yes/no) or breast biopsy (0, 1, 2, 3+ biopsies), first-degree family history of breast cancer (0, 1, 2+ first-degree relatives—mother, daughter, or sister—with a breast cancer diagnosis), first-degree family history of ovarian cancer (0, 1, 2+ first-degree relatives—mother, daughter, or sister—with an ovarian cancer diagnosis), previous gynecologic surgery (defined as hysterectomy and/or partial or bilateral oophorectomy), history of endometriosis (yes/no), history of uterine fibroids (yes/no), use of tobacco (never, former, current smoker; cigarettes per day smoked) and alcohol consumption (drinks/day), and serum CA 125 level at baseline (PLCO only). For PLCO, we tested potential interactions with randomization arm and, for ovarian cancer, method of detection (screen-detected versus not screen-detected). In most instances, the PLCO and NIH-AARP questionnaires allowed for identically coded variables. To synchronize age at menarche (PLCO categories <10, 10–11, 12–13, 14–15, 16+ y; NIH-AARP categories ≤12, 13–14, 15+ y), we randomly allocated women in the overlapping PLCO categories (e.g., 12–13 y) to the NIH-AARP categories (≤12 y or 13–14 y) using the age at menarche distribution from the nationally representative NHANES study [17]. For OC use we randomly assigned women in the ≤1 year category in PLCO to the <1 and 1+ year categories.

Alcohol consumption was not ascertained in the PLCO control arm, and thus RRs for association with alcohol consumption were estimated from the PLCO intervention arm and NIH-AARP. RRs for duration of estrogen and progestin MHT use and duration of unopposed estrogen MHT use were estimated from women in the NIH-AARP cohort who had responded to the second questionnaire. Information for benign breast disease was missing on 20% of the women in the dataset, and we thus created an indicator for missing values. For all other variables, women with missing values were excluded as the number missing was very small (<5% for all variables; Table 5). We first assessed all risk factors listed above as possible predictors for each cancer as main effects. Final models included only variables that were significant in multivariable models with *p*<0.01. We chose a stringent *p*-value as we did not want to include variables with modest RRs that would not improve prediction. Model building was repeated using stepwise variable selection in Cox proportional hazards models and led to the same variables being selected for each cancer. We also assessed the significance at *p*<0.01 of all first-order interaction terms of variables included in the final models. We fitted variables with trends whenever appropriate. For all risk factors, the reference category was the lowest risk category, to facilitate attributable risk (AR) computations. To accommodate missing data, we calculated AR for the breast cancer model in women with complete data. Using time on study as the timescale, adjustment for calendar period or additional censoring at age of diagnosis of any other cancer (e.g., censoring the breast cancer models at diagnosis of endometrial cancer or ovarian cancer) or adjustment for gynecologic surgery did not change the results appreciably.

**Table 5:** Selected characteristics of non-Hispanic, white women in the NIH-AARP and PLCO cohorts.

Characteristic	Subcategory	Breast Cancer		Endometrial Cancer		Ovarian Cancer	
		Cases, *N* (Percent)	Incidence per 100,000 Person-Years^1^	Cases, *N* (Percent)	Incidence per 100,000 Person-Years	Cases, *N* (Percent)	Incidence per 100,000 Person-Years
**All**		8,158	428	1,656	138	881	55
**BMI, kg/m^2^**	<25	3,406 (42)	413	500 (30)	91	404 (46)	57
	25 to <30	2,693 (33)	439	446 (27)	118	269 (31)	52
	30 to <35	1,182 (14)	436	313 (41)	197	109 (12)	49
	35 to <40	478 (6)	483	186 (11)	318	50 (6)	61
	40+	236 (3)	441	176 (11)	539	26 (3)	59
	Missing	163 (2)	381	35 (2)	135	23 (3)	67
**OC use, years**	≤1	4,853 (59)	426	1,155 (70)	161	599 (68)	63
	2–9	2,363 (29)	429	386 (23)	112	223 (25)	48
	10+	875 (11)	443	112 (7)	84	54 (6)	32
	Missing	67 (1)	379	3 (0)	54	5 (1)	68
**MHT use, years**	0	2,838 (35)	373	873 (53)	139	367 (42)	50
	1–9	3,085 (38)	452	480 (29)	111	306 (35)	52
	10+	2,111 (26)	496	285 (17)	235	193 (22)	76
	Missing	124 (2)	342	18 (1)	93	15 (2)	61
**Parity**	Nulliparous	1,234 (15)	510	318 (19)	176	146 (17)	71
	1–2	2,860 (35)	437	586 (35)	139	325 (37)	59
	3+	3,922 (48)	401	728 (44)	125	403 (46)	49
	Missing	142 (2)	485	24 (1)	176	7 (1)	41
**Age at menopause, years**	<50	2,850 (35)	459	781 (47)	141	330 (37)	56
	50–54	4,069 (50)	388	499 (30)	115	436 (49)	55
	55+	876 (11)	570	281 (17)	196	78 (9)	52
	Premenopausal	363 (4)	442	95 (6)	133	37 (4)	50
**Family history of breast or ovarian cancer**	No	6,267 (77)	401	1,359 (82)	138	693 (79)	53
	Yes	1,741 (21)	569	265 (16)	137	177 (20)	68
	Missing	150 (2)	417	32 (2)	157	11 (1)	41
**Cigarette smoking**	Never smoker	3,674 (45)	409	897 (54)	159	449 (51)	59
	Current smoker	1,037 (13)	429	127 (8)	83	97 (11)	48
	Former smoker	3,267 (40)	453	607 (37)	131	323 (37)	53
	Missing	180 (2)	415	25 (2)	111	12 (1)	41
**Alcohol consumption, drinks/day**	0	1,776 (22)	404	392 (24)	156	192 (22)	55
	<1	3,942 (48)	417	838 (51)	141	437 (50)	56
	1+	1,092 (13)	531	157 (9)	113	98 (11)	56
	Missing	1,348 (17)	428	269 (16)	124	154 (17)	52
**Age at birth of first child, years**	<25	4,562 (56)	391	875 (53)	131	494 (56)	51
	25+	2,220 (27)	477	439 (27)	129	234 (27)	56
	NA or missing	1,376 (17)	507	342 (21)	176	153 (17)	69
**History of gynecologic surgery**	Yes	2,953 (36)	382	28 (2)	125	262 (30)	63
	No	4,892 (60)	461	1,567 (95)	139	597 (68)	53
	Missing	313 (4)	439	61 (4)	121	22 (2)	42
**Benign breast disease**	Missing	1,393 (17)	365	285 (17)	127	156 (18)	53
	No	3,355 (41)	376	772 (47)	132	399 (45)	53
	Yes	3,410 (42)	540	599 (36)	152	326 (37)	59

NA, not applicable.

#### Absolute risk models

The absolute risk π(*a*,*b*) of cancer *c* (*c* = breast, endometrial, or ovarian cancer) in the age interval (*a*,*b*] is the probability of developing a specific cancer *c* during that interval, given that one is alive and free of previous cancer *c* at the beginning of the interval. The absolute risk is reduced by death from causes other than cancer *c* and is defined by
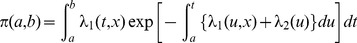
(1) In Equation 1, λ_1_ and λ_2_ are the cause-specific hazard rates for cancer *c* and for competing causes, respectively.

We modeled λ_1_(*a*,*x*) = λ_10_(*a*) rr_1_(*a*,*x*) as the product of the age-specific baseline hazard rate λ_1_(*a*) and the RR model, rr_1_(*a*,*x*) = exp(β'*x*), where *x* denotes covariates. We did not include covariates in the hazard λ_2_ for competing causes of death. The age-specific baseline hazard rates λ_10_(*a*) are computed by multiplying the age-specific SEER incidence rates, λ_1_*(*a*), by one minus the estimate of the AR, i.e., λ_10_(*a*) = λ_1_*(*a*)(1−AR_1_(*a*)), as outlined in [2]. The AR for cancer type *c* was obtained as one minus the number of women in the dataset divided by the sum of the multivariate RR estimates for cancer *c* over all women. We used a model with piecewise constant hazards to approximate Equation 1, with 5-y age intervals.

In SEER, endometrial and ovarian cancer incidence rates are calculated based on all women in the population in a given age group. These rates thus are lower than rates that are based on women with an intact uterus or ovaries, respectively. We therefore adjusted the age-specific SEER rates by dividing them by the percentage of women who had not had a hysterectomy (estimated from the Behavioral Risk Factor Surveillance System (BRFSS) survey [18] for the same areas included in SEER) or oophorectomy (estimated from NHANES [17]) (see Tables 4 and S1, S2, S3). In Table S1 we also present rates for models that allow for the possibility that a woman might have a hysterectomy or an oophorectomy during the projection interval, by adding hysterectomy and oophorectomy rates to the competing risks for the two models, respectively.

#### Statistical analysis used for model validation

We compared the expected and the observed numbers of cases overall and in subgroups defined by risk factor combinations. The expected number of cases was calculated by summing the individual projected probabilities, given the baseline covariate values for each woman from entry (July 1990) into the NHS cohort to June 2004. The 95% CIs for the expected/observed (*E/O*) ratios were calculated using the normal approximation to the Poisson distribution: 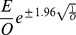. An *E/O* ratio above one indicates that the model overestimates cancer risk, and an *E/O* less than one indicates that the model underestimates cancer risk. We evaluated the discriminatory accuracy of the prediction models using the area under the receiver operating characteristic curve (AUC), with 95% bootstrap CIs.

## Results

The characteristics of the women in the study and cancer incidence rates are shown in Table 1.

### Relative Risk Models

#### Breast cancer

Of the 240,712 women used to fit the final breast cancer RR model, 7,695 were diagnosed with invasive breast cancer. The following variables were included in the final RR model: BMI (<25, 25 to <30, 30 to <35, 35+ kg/m^2^), estrogen and progestin MHT use (never, <10, 10+ y), other MHT use (no, yes), parity (0, 1+ children), age at first birth (<25, 25–29, 30+ y), premenopausal (no, yes), age at menopause (<50, 50 to <55, 55+ y), benign breast diseases (no, yes), family history of breast or ovarian cancer (no, yes), and alcohol consumption (0, <1, 1+ drinks/day). The largest RRs per category increase in the model (Table 6) were obtained for having used estrogen and progestin MHT, RR = 1.40 (95% CI: 1.32–1.49) per category increase in duration, and having a history of benign breast disease/biopsy, RR = 1.40 (95% CI: 1.33–1.48).

**Table 6:** Multivariate relative risk estimates for breast, endometrial, and ovarian cancer among non-Hispanic, white women in the NIH-AARP and PLCO cohorts.

Characteristic	Subcategory	RR (95% CI)		
		Breast Cancer	Endometrial Cancer	Ovarian Cancer
**BMI**	<25 kg/m^2^	1.0 (referent)	1.0 (referent)	—
	Per category increase	1.09 (1.06–1.11)	1.72 (1.65–1.80)	
**OC use**	1+ y	—	1.0 (referent)	1.0 (referent)
	<1 y		1.44 (1.29–1.62)	1.36 (1.17–1.59)
**MHT use**	Never		1.0 (referent)	1.0 (referent)
	Per category increase		1.15 (1.05–1.26)	1.26 (1.15–1.38)
**Interaction MHT use with BMI<25 kg/m^2^**			1.61 (1.43–1.81)	
**Estrogen and progestin MHT use**	No MTH use	1.0 (referent)		
	Per category increase	1.40 (1.32–1.49)		
**Other MHT use**	No	1.0 (referent)		
	Yes	1.16 (1.08–1.25)		
**Age at first live birth**	<25 y	1.0 (referent)	—	—
	Per category increase	1.17 (1.12–1.21)		
**Parity**	3+ children		1.0 (referent)	1.0 (referent)
	Per category increase		1.21 (1.13–1.29)	1.25 (1.14–1.37)
	1+ children	1.0 (referent)		
	Nulliparous	1.32 (1.23–1.40)	—	—
**Age at menopause**	<50 y	1.0 (referent)	1.0 (referent)	—
	Per category increase	1.18 (1.14–1.22)	1.26 (1.17–1.35)	
	NA/missing	1.17 (1.14–1.22)	1.29 (1.01–1.63)	
**Benign breast disease/biopsy**	No	1.0 (referent)		
	Yes	1.40 (1.33–1.48)		
**Family history of breast or ovarian cancer**	No	1.0 (referent)		1.0 (referent)
	Yes	1.39 (1.32–1.47)	—	1.27 (1.07–1.50)
**Alcohol consumption**	0 drinks/day	1.0 (referent)	—	—
	Per category increase	1.12 (1.08–1.17)		
**Smoking**	Current smoker	—	1.0 (referent)	—
	Never smoker		1.47 (1.22–1.78)	
	Former smoker		1.21 (1.00–1.47)	

Categories for variables fitted with a trend: Models are adjusted for randomization group in PLCO and calendar time. All variables are coded so that the lowest risk category is the reference category. BMI: <25, 25 to <30, 30 to <35, and 35+ kg/m^2^ for breast cancer and <25, 25 to <30, 30 to <35, 35 to <40, and 40+ kg/m^2^ for endometrial cancer; MHT use: never, <10 y, 10+ y; estrogen and progestin MHT use: never, <10 y, 10+ y; other/unknown MHT use: no, yes; age at first birth: <25, 25–29, and 30+ y; parity: 0, 1–2, and 3+ children for endometrial and ovarian cancer; age at menopause: <50, 50–54, 55+ y, missing; benign breast diseases/biopsy: no, yes, missing; alcohol consumption: 0, <1, 1+ drinks/day.

NA, not applicable.

#### Endometrial cancer

Of the 146,679 women included in the final endometrial cancer model, 1,559 were diagnosed with endometrial carcinoma. The final RR model included BMI (<25, 25 to <30, 30 to <35, 35 to <40, 40+ kg/m^2^), MHT use (never, <10, 10+ y), parity (0, 1–2, 3+ children), premenopausal (no, yes), age at menopause (<50, 50 to <55, 55+ y), smoking (never, former, current), OC use (<1, 1+ y), and an interaction term between MHT use and an indicator variable that was one for BMI<25 kg/m^2^ and zero otherwise. The largest RRs in the model were obtained for BMI, fitted with a trend RR = 1.72 per category increase (95% CI: 1.65–1.80); being a never smoker, with RR = 1.47 (95% CI: 1.22–1.78) compared to a current smoker; and the interaction term between MHT use and the BMI<25 kg/m^2^ indicator, RR = 1.61 (95% CI: 1.43–1.81) (Table 6).

#### Ovarian cancer

Among the 199,973 women included in the ovarian cancer analysis, 844 were diagnosed with ovarian carcinoma. The final RR model included family history of breast or ovarian cancer (no, yes), duration of MHT use (never, <10, 10+ y), parity (0, 1–2, 3+ children), and OC use (<1, 1+ y) (Table 6). The largest RR was seen for OC never use compared to OC ever use, RR = 1.36 (95% CI: 1.17–1.59).

### Absolute Risks

The AR estimates used to compute baseline hazard rates for the models were 0.52 for breast cancer, 0.81 for endometrial cancer, and 0.43 for ovarian cancer.

Table 7 shows examples of 10- and 20-y absolute risks for several risk profiles and for initial ages 50 and 65 y. Breast cancer absolute risk estimates ranged from 1.57% to 21.78% for 10-y projections and from 3.64% to 35.11% for 20-y projections. Risk for endometrial cancer ranged from 0.35% to 10.50% for 10-y and from 1.22% to 17.08% for 20 y. The highest 20-y risk, 17.08%, was seen for a 65-y-old woman in the 40+ kg/m^2^ BMI category who had never smoked. For specific risk factor combinations, endometrial absolute risk can be higher than breast cancer absolute risk. For example, profile 8 (Table 7) corresponds to a premenopausal nulliparious 50-y-old woman with a BMI of 35 kg/m^2^, who never smoked, has no family history of breast or ovarian cancer, and never had a breast biopsy. Her 10-y endometrial cancer absolute risk is 4.9%, while her 10-y absolute risk of breast cancer is only 1.62%. 10-y absolute risk of ovarian cancer ranged from 0.28% to 0.96%, and 20-y risk, from 0.74% to 1.77%.

**Table 7:** Examples of 10- and 20-y absolute risk estimates for breast, endometrial, and ovarian cancer using SEER rates corrected for hysterectomy for endometrial cancer and for oophorectomy for ovarian cancer in non-Hispanic, white women.

Profile Number	Age (Years)	Smoking	BMI (kg/m^2^)	OC Use	Age at Birth of First Child (Years)	Number of Live Births	Age at Menopause (Years)	Duration of MHT Use (Years)	Duration of Estrogen and Progestin MHT Use (Years)	Other MHT Use	Family History Ovarian/Breast Cancer	Diagnosis of Benign Breast Disease/Biopsy	Alcohol Consumption (Drinks/Day)	10-y/20-y Absolute Cancer Risk (Percent)		
														Breast	Endometrium	Ovary
1	50	N	24	N	25	3	NA	0	0	N	N	N	0	1.83/4.23	0.51/1.36	0.28/0.74
2	50	N	21	N	24	4	NA	0	0	N	N	N	0	1.57/3.64	0.51/1.36	0.28/0.74
3	50	C	24	N	21	3	NA	0	0	N	N	N	<1	1.76/4.07	0.35/0.92	0.28/0.74
4	50	C	25	N	22	3	NA	0	0	Y	N	Y	<1	3.09/7.08	0.59/1.59	0.28/0.74
5	50	F	30	Y	37	1	50	1	1	N	Y	Y	≥1	8.75/19.26	1.17/3.09	0.41/1.09
6	50	F	35	Y	41	1	50	1	1	N	Y	Y	≥1	9.46/20.71	2.00/5.26	0.41/1.09
7	50	N	40	Y	39	1	50	1	1	N	Y	N	≥1	6.84/15.27	4.13/10.67	0.41/1.09
8	50	N	35	Y	NA	0	NA	11	11	N	N	N	≥1	6.37/14.28	3.42/8.91	0.51/1.34
9	65	N	24	N	25	3	49	0	0	N	N	N	0	2.45/4.35	0.72/1.22	0.52/0.96
10	65	F	21	N	24	4	47	3	3	N	N	N	0	2.94/5.21	1.09/1.85	0.65/1.21
112	65	F	24	N	21	3	49	5	5	N	N	N	<1	3.29/5.83	1.09/1.85	0.65/1.21
121	65	C	25	N	22	3	48	5	5	N	N	Y	<1	4.96/8.71	0.97/1.64	0.65/1.21
132	65	C	30	Y	37	1	55	10	10	N	Y	Y	≥1	20.27/32.94	2.52/4.25	0.96/1.77
143	65	F	35	Y	41	1	57	11	11	N	Y	Y	≥1	21.78/35.11	5.18/8.62	0.96/1.77
15	65	N	40	Y	39	1	56	12	12	N	Y	N	≥1	16.09/26.73	10.50/17.08	0.96/1.77

C, current; F, former; N, no; NA, not applicable; Y, yes.

Comprehensive tabulations of 5-, 10-, and 20-y risks for 50- and 60-y-old women for all possible combinations of risk factors for the three outcomes, and software written in SAS to compute absolute risk estimates for any age, projection length, and combination of risk factors, are freely available for download under Breast/Endometrial/Ovarian Risk Assessment at http://dceg.cancer.gov/tools/risk-assessment.

### Validation of the Models in the Nurses' Health Study

RR estimates in the NHS cohort for all three cancers (Table 8) were very similar to those used in the model (Table 6).

**Table 8:** Multivariate relative risk estimates for breast, endometrial, and ovarian cancer among non-Hispanic, white women in the NHS validation cohort.

Characteristic	Subcategory	RR (95% CI)		
		Breast Cancer	Endometrial Cancer	Ovarian Cancer
**BMI**	<25 kg/m^2^	1.0 (referent)	1.0 (referent)	—
	Per category increase	1.11 (1.07–1.16)	1.78 (1.64–1.93)	—
**OC use**	1+ y	—	1.0 (referent)	1.0 (referent)
	<1 y	—	1.30 (1.07–1.57)	1.36 (1.08–1.73)
**Estrogen and progestin MHT use**	No MTH use	1.0 (referent)	—	—
	Per category increase	1.53 (1.37–1.70)	—	—
**Other MHT use**	No	1.0 (referent)	—	—
	Yes	1.07 (0.98–1.17)	—	—
**MHT use**	Never	—	1.0 (referent)	1.0 (referent)
	Per category increase	—	2.43 (1.97–2.99)	1.24 (1.05–1.46)
**Interaction MHT use with BMI<25 kg/m^2^**			1.41 (1.10–1.80)	
**Age at first live birth**	<25 y	1.0 (referent)	—	—
	Per category increase	1.14 (1.08–1.20)	—	—
**Parity**	3+ children	—	1.0 (referent)	1.0 (referent)
	Per category increase	—	1.26 (1.11–1.44)	1.26 (1.08–1.47)
	1+ children	1.0 (referent)	—	—
	Nulliparous	1.31 (1.13–1.52)	—	—
**Age at menopause**	<50	1.0 (referent)	1.0 (referent)	—
	Per category increase	1.20 (1.12–1.29)	1.30 (1.12–1.51)	—
	Premenopausal	1.54 (1.32–1.80)	2.16 (1.57–2.97)	—
**Benign breast disease/biopsy**	No	1.0 (referent)	—	—
	Yes	1.34 (1.24–1.44)	—	—
**Family history of breast or ovarian cancer**	No	1.0 (referent)	—	1.0 (referent)
	Yes	1.34 (1.22–1.47)	—	1.29 (0.98–1.71)
**Alcohol consumption**	0 drinks/day	1.0 (referent)	—	—
	Per category increase	1.06 (1.00–1.12)	—	—
**Smoking**	Current smoker	—	1.0 (referent)	—
	Never smoker	—	1.82 (1.36–2.44)	—
	Former smoker	—	1.30 (0.96–1.76)	—

Categories for variables fitted with a trend: All variables are coded so that the lowest risk category is the reference category. BMI: <25, 25 to <30, 30 to <35, and 35+ kg/m^2^ for breast cancer and <25, 25 to <30, 30 to <35, 35 to <40, and 40+ kg/m^2^ for endometrial cancer; estrogen and progestin MHT use: never, <10 y, 10+ y; other/unknown MHT use: no, yes; MHT use: never, <10 y, 10+ y; age at first birth: <25, 25–29, and 30+ y; parity: 0, 1–2, and 3+ children for endometrial and ovarian cancer; age at menopause: <50, 50–54, 55+ y, missing; benign breast disease/biopsy: no, yes, missing; alcohol consumption: 0, <1, 1+ drinks/day.

#### Breast cancer

A total of 2,934 incident breast cancers were diagnosed among women included in the analyses, and the model predicted 2,930, resulting in an *E/O* ratio of 1.00 (95% CI: 0.96–1.04) (Table 9). The model significantly underestimated the number of breast cancers in premenopausal women. For all other variables, the *E/O* ratios were not statistically significantly different from unity. The overall AUC (discriminatory power) in the NHS cohort was 0.58 (95% CI: 0.57–0.59). We also compared the performance of the new breast cancer model to that of NCI's BCRAT [6] on the same validation data. BCRAT predicted 2,947 cases, resulting in *E/O* = 1.00 (95% CI: 0.97–1.04). The number of cases was overpredicted significantly in cells defined by family history using either the coding for the new model or BCRAT (Table 9). For women with a family history of breast or ovarian cancer, *E/O* = 1.30 (95% CI: 1.19–1.42), and for women with one and two relatives with breast cancer, *E/O* = 1.30 (95% CI: 1.18–1.43) and *E/O* = 1.78 (95% CI: 1.25–2.55), respectively. For the new breast cancer model, the predicted number of cases did not significantly differ from the observed, with *E/O* = 0.98 (95% CI: 0.89–1.08) for women with one first-degree relative with breast cancer and *E/O* = 0.72 (95% CI: 0.50–1.02) for women with two or more relatives with breast cancer. The overall AUC for BCRAT in the NHS validation data, 0.56 (95% CI: 0.55–0.58), was significantly lower than the AUC for the new breast cancer model (paired *t*-test, *p*<0.001).

**Table 9:** Breast cancer risk predictions during the follow-up of non-Hispanic, white women in the NHS for new breast cancer model and BCRAT.

Characteristic	Observed	Breast Cancer Model		BCRAT	
		Expected	*E/O* (95% CI)	Expected	*E/O* (95% CI)
**All**	2,934	2,930	1.00 (0.96–1.04)	2,947	1.00 (0.97–1.04)
**BMI**					
<25 kg/m^2^	1,447	1,492	1.03 (0.98–1.09)	1,545	1.07 (1.01–1.12)
25 to <30 kg/m^2^	971	935	0.96 (0.90–1.03)	930	0.96 (0.90–1.02)
30 to <35 kg/m^2^	368	349	0.95 (0.86–1.05)	329	0.89 (0.81–0.99)
35 to <40 kg/m^2^	148	154	1.04 (0.89–1.22)	139	0.94 (0.80–1.11)
**Duration of estrogen and progestin MHT use**					
0	2,367	2,402	1.01 (0.97–1.06)	2,517	1.06 (1.02–1.11)
1–9 y	567	527	0.93 (0.86–1.01)	426	0.75 (0.69–0.82)
10+ y	0	2	NA	1	NA
**Other MHT use**					
No	1,746	1,640	0.94 (0.90–0.98)	1,667	0.95 (0.91–1.00)
Yes	1,188	1,290	1.09 (1.03–1.15)	1,277	1.07 (1.02–1.14)
**Parity**					
0	197	201	1.02 (0.89–1.17)	136	0.69 (0.60–0.79)
1+	2,737	2,729	1.00 (0.96–1.04)	2,807	1.03 (0.99–1.06)
**Age at menarche**					
<12 y	654	623	0.95 (0.88–1.03)	686	1.05 (0.97–1.13)
12–13 y	1,636	1,656	1.01 (0.96–1.06)	1,680	1.03 (0.98–1.08)
≥14 y	613	624	1.02 (0.94–1.10)	581	0.95 (0.88–1.03)
**Age at first birth**					
<25 y or nulliparous	1,601	1,586	0.99 (0.94–1.04)	1,586	0.99 (0.94–1.04)
25 to <30 y	1,024	1,021	1.00 (0.94–1.06)	1,063	1.04 (0.98–1.10)
30+ y	309	323	1.05 (0.94–1.17)	347	1.12 (1.00–1.26)
**Age at menopause**					
<50 y	866	911	1.05 (0.98–1.12)		
50–54 y	1,605	1,634	1.02 (0.97–1.07)	1,574	0.98 (0.93–1.03)
55+ y	196	189	0.96 (0.84–1.11)	157	0.80 (0.70–0.92)
**Premenopausal**	267	196	0.73 (0.65–0.83)	217	0.81 (0.72–0.92)
**Benign breast disease**					
No	1,548	1,517	0.98 (0.93–1.03)	1,863	1.00 (0.95–1.04)
Yes	1,386	1,413	1.02 (0.97–1.07)	1,083	1.02 (0.96–1.09)
**Family history of breast or ovarian cancer**					
No	2,410	2,387	0.99 (0.95–1.03)	2,262	0.94 (0.90–0.98)
Yes	524	543	1.04 (0.95–1.13)	682	1.30 (1.19–1.42)
**Number of first-degree relatives with breast cancer**					
0	2,464	2,475	1.00 (0.97–1.05)	2,328	0.94 (0.91–0.98)
1	435	428	0.98 (0.89–1.08)	565	1.30 (1.18–1.43)
2	30	21	0.72 (0.50–1.02)	53	1.78 (1.25–2.55)
**Alcohol consumption**					
0 drinks/day	1,139	1,091	0.96 (0.90–1.02)	1,185	1.04 (0.98–1.10)
<1 drinks/day	1,389	1,418	1.02 (0.97–1.08)	1,388	1.00 (0.95–1.05)
1+ drinks/day	406	421	1.04 (0.94–1.14)	370	0.91 (0.83–1.00)

NA, not applicable.

#### Endometrial cancer

The endometrial cancer absolute risk model predicted 640 cancers, but only 532 incident endometrial cancers were observed, resulting in *E/O* = 1.20 (95% CI: 1.11–1.30). The model significantly overestimated the observed NHS endometrial cancers in most subgroups (Table 10). The number of endometrial cancers was underestimated for women in the highest BMI category and for premenopausal women, and significantly underestimated for women who reported taking MHT for 10 or more years. The AUC was 0.68 (95% CI: 0.66–0.70).

**Table 10:** Endometrial and ovarian cancer risk prediction during the follow-up of non-Hispanic, white women in the NHS.

Characteristic	Endometrial Cancer			Ovarian Cancer		
	Observed	Expected	*E/O* (95% CI)	Observed	Expected	*E/O* (95% CI)
**All**	532	637	1.20 (1.11–1.29)	377	406	1.08 (0.97–1.19)
**BMI**						
<25 kg/m^2^	190	235	1.24 (1.09–1.40)	—	—	—
25 to <30 kg/m^2^	160	185	1.16 (1.00–1.34)	—	—	—
30 to <35 kg/m^2^	96	117	1.22 (1.02–1.46)	—	—	—
35 to <40 kg/m^2^	42	57	1.37 (1.06–1.77)	—	—	—
40+ kg/m^2^	44	42	0.95 (0.70–1.29)	—	—	—
**Duration of MHT use**						
0 y	304	388	1.28 (1.15–1.41)	202	226	1.12 (0.97–1.28)
1–9 y	186	228	1.23 (1.08–1.40)	143	154	1.08 (0.92–1.27)
10+ y	42	21	0.49 (0.32–0.76)	32	26	0.80 (0.57–1.13)
**Parity**						
0	43	52	1.20 (0.91–1.58)	36	33	0.91 (0.66–1.26)
1–2	190	211	1.11 (0.97–1.27)	120	137	1.14 (0.95–1.36)
3+	299	374	1.25 (1.13–1.38)	221	236	1.07 (0.94–1.22)
**Age at menopause**						
<50 y	123	172	1.39 (1.20–1.62)	—	—	—
50–54 y	291	338	1.16 (1.04–1.29)	—	—	—
55+ y	41	63	1.53 (1.20–1.96)	—	—	—
Premenopausal	77	65	0.84 (0.66–1.07)			
**Family history of breast or ovarian cancer**						
No	—	—		318	344	1.08 (0.97–1.21)
Yes	—	—		59	62	1.04 (0.81–1.35)
**OC use**						
Never or <1 y	367	459	1.25 (1.14–1.37)	270	291	1.08 (0.96–1.21)
1+ y	165	178	1.08 (0.93–1.25)	107	115	1.07 (0.89–1.30)
**Smoking**						
Never	287	318	1.11 (0.99–1.24)	—	—	—
Former	191	243	1.27 (1.12–1.44)	—	—	—
Current	54	76	1.40 (1.12–1.75)	—	—	—

#### Ovarian cancer

A total of 377 incident ovarian cancers were diagnosed among women included in the analyses, and the model predicted 406 (Table 10), resulting in *E/O* = 1.08 (0.97–1.19). The number of ovarian cancers was overestimated for all covariate categories, albeit not statistically significantly, with the exception of the nulliparous group and women taking MHT for 10 or more years. The AUC was 0.59 (95% CI: 0.56–0.63).

### Cross-Classification of Breast and Endometrial Cancer Risk in the Nurses' Health Study

Current American Society of Clinical Oncology guidelines indicate that premenopausal women and postmenopausal women with low risk of side effects and a 5-y projected BCRAT risk ≥1.66% may benefit from tamoxifen and/or raloxifene for breast cancer prevention [11]. Among the 3,837 premenopausal women aged 50–55 y at baseline in the NHS cohort who reported having a uterus during follow-up, 784 had a 5-y absolute breast cancer risk ≥1.66%. Of those, three women had a ≥2% 5-y absolute endometrial cancer risk. Tamoxifen reduces breast cancer risk by approximately 50% and increases endometrial cancer risk approximately 4-fold in older women [9]. Gail et al. [19] and Freedman et al. [20] weighed the risks and benefits of tamoxifen assuming average age-specific risks of health outcomes, apart from breast cancer. Using models both for breast cancer and for endometrial cancer yields a more accurate weighing of risks and benefits. For example, based on an average 5-y risk of endometrial cancer of 0.41% [19], tamoxifen would increase absolute endometrial cancer risk by 1.23% while reducing a 2.5% breast cancer risk by 1.25%. These risks and benefits are nearly equal. If, instead, the woman had an endometrial cancer risk of 2%, tamoxifen would increase endometrial cancer risk by 6%, which greatly exceeds the reduction in breast cancer risk. In carefully balancing risk and benefits, however, the comparative lethality of the two outcomes might also be an additional important factor in a decision concerning whether to take tamoxifen.

## Discussion

We developed models that predict individualized probabilities of developing breast, endometrial, and ovarian cancers among US white women aged 50+ y. We chose these three cancers because they share several risk factors, presumably reflecting a common hormonal etiology, and because management decisions may depend jointly on these risks.

To our knowledge, there is no other absolute risk model for endometrial cancer, despite the fact that it is the fourth most common cancer in women [8] and its absolute risks are quite high, particularly in obese women (Table 7). Knowledge of endometrial cancer risk might inform decision-making about diagnostic workup, clinical management, surgical interventions, and the use of agents, such as tamoxifen or unopposed estrogen that increase endometrial cancer risk. Such a model might also aid in designing intervention trials to prevent endometrial cancer and in identifying women with elevated risk who might benefit from such interventions. The endometrial cancer model may also be useful in assessing the burden of that cancer in the general population, which may increase, as more than a third of all US women now have a BMI of 30 kg/m^2^ or higher [21]. In combination with data on trends in the prevalence of obesity, one could use the model to investigate the extent to which the increasing prevalence of obesity accounts for the significant 2% per year increase in endometrial cancer incidence seen among white women 2006–2010 [22].

Unlike most other models for breast cancer, our model includes factors that are potentially modifiable for the individual or in populations over time, such as alcohol consumption, BMI, and use of MHT. This model had slightly better discriminatory accuracy (AUC = 0.58) in the validation data than the widely used BCRAT (“Gail model”) (AUC = 0.56), which predicts breast cancer risk based on reproductive factors, number of breast biopsies, and atypical hyperplasia. However, despite this increase in AUC, the discriminatory accuracy of the breast cancer absolute risk model is still modest, and limits its clinical applicability, particularly for screening. Several breast cancer risk prediction models include non-modifiable risk factors such as family history (e.g., [1]), mammographic density [23]–[25], and reproductive and medical factors such as age at menarche and number of breast biopsies [6]. A few models for US women include potentially modifiable risk factors, such as BMI and alcohol use [26]. Our model also incorporates use, and duration of use, of combined estrogen and progestin MHT and other types of MHT. Use of estrogen and progestin MHT had the second largest RR in our model, and had the same RR (RR = 1.40) for a one category increase in duration as benign breast disease. Petracci et al. [27] illustrate public health and counseling applications of a breast cancer model with modifiable risk factors in Italian women, and similar calculations could be performed for the US with the model we developed. These calculations could also aid in understanding the impact of increases in obesity on US breast cancer incidence.

For ovarian cancer, a model based on the NHS includes age at menopause, age at menarche, OC use, and tubal ligation [7].

To build the models, we combined data from two large prospective studies from well-characterized populations. We used another large cohort, the NHS cohort, to independently validate our models. The RR estimates agreed well with those in the NHS cohort for all three models. The discriminatory power, as assessed by the AUC, was 0.58 and 0.59 for the breast and ovarian cancer models, respectively. While these values indicate modest discriminatory ability, they are similar to those reported for other cancer risk models for these cancers [2],[7]. The AUC value for endometrial cancer was 0.67, which is larger than that seen for most models of cancer incidence.

The breast and ovarian model were well calibrated in the NHS cohort; however, because women in the NHS cohort were censored at the age of diagnosis of an in situ breast cancer, the breast model may have underestimated slightly. The endometrial model significantly over-predicted the number of endometrial cancers. This reflects the fact that the NHS cohort has considerably lower endometrial cancer rates (Table 3) than those seen in SEER (Table 4). Further studies of the calibration of this model in additional cohorts would be desirable to assess its applicability to the general US population.

Well-calibrated risk models, even those with modest discriminatory accuracy, have several public health applications. These include designing cancer prevention trials, assessing the absolute burden of disease in the population and in subgroups, and gauging the potential absolute reductions in risk from preventive strategies. Using risk models to select individuals for screening or other interventions usually requires high discriminatory accuracy [28]. Well-calibrated risk models with modest discriminatory accuracy can also aid in individual decision-making. Such models can provide realistic information on level of risk that is useful in making decisions, such as whether or not to have a mammogram [29]. Such models are also useful in decisions on whether or not to take an intervention that has both beneficial and harmful health effects [19],[28].

There are several potential limitations to our models. The exact number of breast biopsies, an important predictor in BCRAT, was not available in our cohorts, and the models were restricted to women aged 50+ y. Our RR models were built using white, non-Hispanic women and may not generalize to other races or ethnicities. We adjusted the age-specific endometrial and ovarian cancer incidence rates for the prevalence of hysterectomy and oophorectomy among US women estimated from population-based surveys, but some residual error may exist. Another limitation is that MHT use was probably more prevalent during the period of study of PLCO and NIH-AARP, from 1993 to 2005, than it is currently. While such changes in the prevalence of risk factors would not affect model calibration, they could reduce the variation of risk in the population and hence reduce the discriminatory accuracy of the models. Among the strengths of our models are large study sample size, nearly complete end point ascertainment, and information on most of the important risk factors.

Our models are not intended to predict the probability of the three cancers among women known to be at much higher than average risk, e.g., women with a mutation in *BRCA1* or *BRCA2* or with hereditary non-polyposis colorectal cancer (HNPCC). Each model is applicable to women without a prior diagnosis of that particular cancer, and thus in principle the breast cancer model can be applied to predict breast cancer risk for women with a prior diagnosis of any other cancer, including endometrial cancer. However, in applying the risk estimates, one needs to consider that a woman's risk may be altered by treatment for a previous cancer.

In conclusion, we developed and assessed models that project the probabilities of developing breast, endometrial, or ovarian cancer among white, non-Hispanic women aged 50+ y. These models might improve the ability to identify potential participants for research studies and assist in clinical decision-making related to the risks of these cancers.

## Supporting Information

Table S1 **Competing mortality rates per 100,000, 1992–2006, from causes other than breast, endometrial, or ovarian cancers obtained from US mortality data for white women.** These data are collected by the National Center for Health Statistics.
(DOCX)

Table S2 **Estimates from the Behavioral Risk Factor Surveillance System for percent of women with hysterectomy in SEER 13 areas, white women, 5-y age groups, 1992–2007 (2001 excluded).**
(DOCX)

Table S3 **Estimates from the NHANES 1999–2000 survey for percent of women with bilateral oophorectomy, white women, 5-y age groups.**
(DOCX)

## Abbreviations

CI: confidence interval
AR: attributable risk
AUC: area under the receiver operating characteristic curve
BCRAT: Breast Cancer Risk Assessment Tool
BMI: body mass index
*E/O*: expected/observed
MHT: menopausal hormone therapy
NCI: National Cancer Institute
NHS: Nurses' Health Study
NIH-AARP: National Institutes of Health–AARP Diet and Health Study
OC: oral contraceptive
PLCO: Prostate, Lung, Colorectal, and Ovarian Cancer Screening Trial
RR: relative risk: SEER, Surveillance, Epidemiology, and End Results Program
