# Supplementary material for: Risk Prediction for Breast, Endometrial, and Ovarian Cancer in White Women Aged 50 y or Older: Derivation and Validation from Population-Based Cohort Studies
Source: PLoS Med. 2013 Jul 30;10(7):e1001492. doi: 10.1371/journal.pmed.1001492 (PMC3728034; doi:10.1371/journal.pmed.1001492)
Supplement: Table S1 — Competing mortality rates per 100,000, 1992–2006, from causes other than breast, endometrial, or ovarian cancers obtained from US mortality data for white women. These data are collected by the National Center for Health Statistics. (DOCX) [file pmed.1001492.s001.docx]

**Table S1: Competing mortality rates per 100,000, 1992-2006, from causes other than breast, endometrial, or ovarian cancers were obtained from US mortality data for white women. These data are collected by the National Center for Health Statistics (NCHS)**

| ***Age group*** | ***All causes-Breast*** | ***All causes other than Corpus and Uterus, NOS*** | ***All causes other than Corpus and Uterus, NOS, including hysterectomy*** | ***All causes other than Ovary*** | ***All causes other than Ovary including bilateral oophorectomy*** |
| --- | --- | --- | --- | --- | --- |
| **50-54** | 290.28 | 327.84 | 1151.61 | 319.66 | 753.03 |
| **55-59** | 463.89 | 512.38 | 2239.44 | 500.84 | 502.27 |
| **60-64** | 789.68 | 851.81 | 2565.72 | 833.8 | 2701.97 |
| **65-69** | 1290.72 | 1365.84 | 1647.53 | 1344.93 | 2424.73 |
| **70-74** | 2106.32 | 2196.04 | 2367.71 | 2170.3 | 2170.30 |
| **75-79** | 3394.22 | 3497.59 | 3685.56 | 3468.23 | 4249.21 |
| **80-84** | 5795.06 | 5913.99 | 5913.99 | 5882.16 | 5919.80 |
| **85+** | 13806.49 | 13961.95 | 13961.95 | 13939.19 | 13939.19 |
