# Supplementary material for: Risk Prediction for Breast, Endometrial, and Ovarian Cancer in White Women Aged 50 y or Older: Derivation and Validation from Population-Based Cohort Studies
Source: PLoS Med. 2013 Jul 30;10(7):e1001492. doi: 10.1371/journal.pmed.1001492 (PMC3728034; doi:10.1371/journal.pmed.1001492)
Supplement: Table S2 — Estimates from the Behavioral Risk Factor Surveillance System for percent of women with hysterectomy in SEER 13 areas, white women, 5-y age groups, 1992–2007 (2001 excluded). (DOCX) [file pmed.1001492.s002.docx]

**Table S2. Estimates from the BRFSS for percent of women with hysterectomy in SEER 13 areas, white women, 5-year age groups, 1992-2007 (2001 excluded)**

| **Age group** | **Had hyst - Yes %** | **Had hyst - No %** | **Don’t know %** | **Refused %** | **Total (N)**** | **Had hyst - Yes (n)** | **Had hyst No- (n)** | **Don’t know (n)** | **Refused (n)** |
| --- | --- | --- | --- | --- | --- | --- | --- | --- | --- |
| **50-54** | 33.31 | 66.24 | 0.06 | 0.39 | 78044 | 25678 | 52106 | 39 | 221 |
| **55-59** | 38.91 | 60.76 | 0.04 | 0.29 | 69785 | 27320 | 42264 | 27 | 174 |
| **60-64** | 43.75 | 55.77 | 0.08 | 0.40 | 61804 | 27360 | 34242 | 28 | 174 |
| **65-69** | 44.54 | 54.99 | 0.06 | 0.42 | 61295 | 28297 | 32737 | 36 | 225 |
| **70-74** | 44.87 | 54.52 | 0.10 | 0.51 | 56703 | 26551 | 29855 | 65 | 232 |
| **75-79** | 45.12 | 54.01 | 0.20 | 0.68 | 49071 | 22824 | 25921 | 77 | 249 |
| **80-84** | 42.57 | 56.14 | 0.26 | 1.03 | 34681 | 15568 | 18816 | 71 | 226 |
| **85+** | 39.55 | 58.65 | 0.54 | 1.25 | 21465 | 8858 | 12303 | 111 | 193 |
