# Supplementary material for: Risk Prediction for Breast, Endometrial, and Ovarian Cancer in White Women Aged 50 y or Older: Derivation and Validation from Population-Based Cohort Studies
Source: PLoS Med. 2013 Jul 30;10(7):e1001492. doi: 10.1371/journal.pmed.1001492 (PMC3728034; doi:10.1371/journal.pmed.1001492)
Supplement: Table S3 — Estimates from the NHANES 1999–2000 survey for percent of women with bilateral oophorectomy, white women, 5-y age groups. (DOCX) [file pmed.1001492.s003.docx]

**Table S3: Estimates from the NHANES 1999-2000 survey for percent (%) of women with bilateral oophorectomy, white women, 5-year age groups**

| **Age group** | **% of women with bilateral oophorectomy** | **Weighted number of women with bilateral oophorectomy.both.percent** | **Weighted Total (N)** |
| --- | --- | --- | --- |
| **50-54** | 19.48 | 1356767 | 6964368 |
| **55-59** | 19.49 | 944617 | 4847344 |
| **60-64** | 26.67 | 1179164 | 4421754 |
| **65-69** | 30.52 | 1208611 | 3959855 |
| **70-74** | 23.21 | 876704 | 3776764 |
| **75-79** | 26.15 | 872098 | 3334504 |
| **80-84** | 26.29 | 678910 | 2582133 |
| **85-99** | 16.11 | 248958 | 1544984 |
